# Cyclobenzaprine Raises ROS Levels in *Leishmania infantum* and Reduces Parasite Burden in Infected Mice

**DOI:** 10.1371/journal.pntd.0005281

**Published:** 2017-01-03

**Authors:** Edézio Ferreira Cunha-Júnior, Valter Viana Andrade-Neto, Marta Lopes Lima, Thais Alves da Costa-Silva, Andres J. Galisteo Junior, Maria A. Abengózar, Coral Barbas, Luis Rivas, Elmo Eduardo Almeida-Amaral, Andre Gustavo Tempone, Eduardo Caio Torres-Santos

**Affiliations:** 1 Laboratório de Bioquímica de Tripanosomatídeos, Instituto Oswaldo Cruz, Fiocruz, Rio de Janeiro, Brasil; 2 Instituto de Medicina Tropical, Universidade de São Paulo, São Paulo, São Paulo, Brazil; 3 Centro de Parasitologia e Micologia, Instituto Adolfo Lutz, São Paulo, São Paulo, Brazil; 4 Centro de Investigaciones Biológicas (CSIC), Unidad Asociada Interacciones, Metabolismo y Bioanálisis CSIC-CEU, Madrid, Spain; 5 Center for Metabolomics and Bioanalysis (CEMBIO), Faculty of Pharmacy, Universidad CEU San Pablo, Madrid, Spain; Northeastern University, UNITED STATES

## Abstract

**Background:**

The leishmanicidal action of tricyclic antidepressants has been studied and evidences have pointed that their action is linked to inhibition of trypanothione reductase, a key enzyme in the redox metabolism of pathogenic trypanosomes. Cyclobenzaprine (CBP) is a tricyclic structurally related to the antidepressant amitriptyline, differing only by the presence of a double bond in the central ring. This paper describes the effect of CBP in experimental visceral leishmaniasis, its inhibitory effect in trypanothione reductase and the potential immunomodulatory activity.

**Methodology/Principal Findings:**

In vitro antileishmanial activity was determined in promastigotes and in *L*. *infantum*-infected macrophages. For *in vivo* studies, *L*. *infantum*-infected BALB/c mice were treated with CBP by oral gavage for five days and the parasite load was estimated. Trypanothione reductase activity was assessed in the soluble fraction of promastigotes of *L*. *infantum*. For evaluation of cytokines, *L*. *infantum*-infected macrophages were co-cultured with BALB/c splenocytes and treated with CBP for 48 h. The supernatant was analyzed for IL-6, IL-10, MCP-1, IFN-γ and TNF-α. CBP demonstrated an IC_50_ of 14.5±1.1μM and an IC_90_ of 74.5±1.2 μM in promastigotes and an IC_50_ of 12.6±1.05 μM and an IC_90_ of 28.7±1.3 μM in intracellular amastigotes. CBP also reduced the parasite load in *L*. *infantum-*infected mice by 40.4±10.3% and 66.7±10.5% in spleen at 24.64 and 49.28 mg/kg, respectively and by 85.6±5.0 and 89.3±4.8% in liver at 24.64 and 49.28mg/kg, after a short-term treatment. CBP inhibited the trypanothione reductase activity with a Ki of 86 ± 7.7 μM and increased the ROS production in promastigotes. CBP inhibited in 53% the production of IL-6 in infected macrophages co-culture.

**Conclusion/Significance:**

To the best of our knowledge, this study is the first report of the *in vivo* antileishmanial activity of the FDA-approved drug CBP. Modulation of immune response and induction of oxidative stress in parasite seem to contribute to this efficacy.

## Author Summary

Leishmaniasis is the denomination of a worldwide widespread group of tropical neglected diseases caused by protozoa belonging to *Leishmania* genus. Several factors have competed to worsen its spreading, such as climate changes and the recent conflict in Syria. The visceral form is the most severe among them and it is fatal if untreated. There is no vaccine for humans and the therapeutic arsenal is quite limited. Drug repurposing is a strategy that aims to look for new uses for well stablished drugs. This approach has been pointed as an attractive option to fasten the development of new treatment for leishmaniasis. Here, the authors evaluated the activity of cyclobenzaprine (CBP), a classical muscle relaxant, in a murine model of visceral leishmaniasis and investigated mechanisms involved in its leishmanicidal action. CBP was effective in reducing the parasite burden both in cell culture and in the liver and spleen of *Leishmania*-infected mice. Furthermore, the experimental data suggested that CBP interferes in the redox balance of the parasite and modulates the immune response of the host. Altogether, these results encourage further studies to evaluate the therapeutic usage of cyclobenzaprine for leishmaniasis.

## Introduction

The research and development of new drugs is a slow and costly process. In addition to the different clinical forms caused by over 20 species of *Leishmania*, the drug resistance increases the complexity of the challenge [1]. Drug repurposing is an interesting strategy to speed up and reduce costs in Drug Discovery and Development [2]. Since the 1980s, the antileishmanial action of tricyclic drugs has been studied [3] and there are evidences that their action is linked to inhibition of trypanothione reductase [4], a key enzyme in the redox metabolism of pathogenic trypanosomes [5]. Cyclobenzaprine is a skeletal muscle relaxant, structurally related to the tricyclic compound amitriptyline, differing only by the presence of a double bond in the central ring. Within this context, we demonstrated the effect of cyclobenzaprine in experimental visceral leishmaniasis, and studied the potential inhibitory effect in trypanothione reductase and also the immunomodulatory activity.

## Materials and Methods

### Ethics statement

This study was performed in accordance with the guidelines of the Guide for the Care and Use of Laboratory Animals of the Brazilian National Council of Animal Experimentation (COBEA) and had the approval of the Animal Ethics Committee of Oswaldo Cruz Foundation (license number L-026/2015).

### Parasites

The *Leishmania infantum* (MHOM/MA/67/ITMAP-263) used in this study is a well-established strain and was kindly provided by Instituto de Biologia Molecular e Celular, Porto University, Portugal.

### Chemicals

Cyclobenzaprine (CBP), DTNB (Ellman's reagent), propidium iodide (PI), resazurin, lipopolysaccharide (LPS), concanavalin A (ConA), miltefosine and rhodamine 123 were purchased from Sigma–Aldrich (St Louis, USA), 2′,7′-dichlorodihydrofluorescein diacetate (H_2_DCFDA) from Molecular Probes (Eugene, USA), and Glucantime from Sanofi-Aventis (São Paulo, Brazil).

### *In vitro* antileishmanial assays

Promastigotes of *L*. *infantum* (strain MHOM/MA/67/ITMAP-263) at 1.0x10^6^ cells/mL were cultivated with cyclobenzaprine in triplicate (0–50 μM) at 26°C in Schneider’s medium (Sigma-Aldrich) supplemented with 20% heat-inactivated fetal calf serum (HIFCS), for 72 h. Inhibition of parasite growth was assessed employing resazurin [6].

For the intracellular amastigote assay, resident peritoneal macrophages were collected from BALB/c mice, plated in RPMI (Sigma-Aldrich) at 2x10^6^ cells/mL in duplicate, infected with promastigotes of *L*. *infantum* (5:1) and incubated at 37°C in 5% CO_2_ for 4 h. After washing, macrophages were incubated with cyclobenzaprine for further 72 h. The slides were stained and the amastigotes were counted using light microscopy.

### Experimental visceral leishmaniasis

*Dose Translation*: The initial dose was calculated based on the human dose, as follows [7]: Animal dose (mg/kg) = (human *K*_*m*_ / animal *K*_*m*_) x human dose (mg/kg), where mouse *K*_*m*_ = 3, human *K*_*m*_ = 37.

*Protocol*: BALB/c mice (five per group) were infected intraperitoneally with 1.0x10^8^ stationary-phase *L*. *infantum* promastigotes [8–10]. After 25 days, mice were treated with three CBP doses (12.32, 24.64 and 49.28 mg/kg) by oral gavage for five days [11]. Control groups were treated with miltefosine 20.55 mg/kg and vehicle (water). After the treatment, the animals were euthanized and spleen and liver were aseptically removed and the parasite load was estimated by limiting dilution assay (LDA) [12]. The serum levels of aspartate aminotransferase (AST) and alanine aminotransferase (ALT) were measured using laboratory colorimetric diagnostic kits (Doles, Goiânia, Brazil).

### Trypanothione reductase activity

The assay was adapted from Hamilton *et al*. [13]. Briefly, promastigotes of *L*. *infantum* were centrifuged and resuspended in buffer containing 40 mM HEPES, 1 mM EDTA and protease inhibitor cocktail (Sigma-Aldrich). The parasites were lysed and centrifuged at 17.500*g*/15 min. The supernatant was considered to contain the trypanothione reductase (TryR). The assay was performed in triplicate with: 40 mM HEPES (pH 7.5), 1 mM EDTA, 100 μM of DTNB (Ellman's reagent), 1 μM substrate (trypanothione trifluoroacetate, Sigma-Aldrich), 0.1 mM NADPH and different concentrations of cyclobenzaprine (0–100μM). Reading was initiated after adding 4 mg/mL soluble protein (enzyme) at 410nm using a Spectra Max M2 spectrofluorometer (Molecular Devices, Silicon Valley, CA, USA) at 10 min intervals for 1 h. Clomipramine was used as positive control (PC). This drug has well-known action on the inhibition of trypanothione reductase enzyme [14]. Apparent Ki was assumed to be the value of the 50% inhibitory concentration obtained from the dose-response curve inhibition of TryR by CBP in 60 minutes. Ki value was determined by nonlinear least-squares fitting of these data to the 4-parameter logistic plot equation, as described by Holloway GA, Charman WN, Fairlamb AH, *et al*, (2009): y = A+{(B–A)/[1+(C/x)^D^]} [15].

### Measurement of ROS

Intracellular ROS levels were measured as described by Ribeiro et al [16]. Briefly, 1×10^7^ promastigotes/mL of *L*. *infantum* were incubated with CBP (0 to 50 μM) in the presence of 20 μM H_2_DCFDA in Schneider’s medium with 20% HIFCS. The fluorescence was quantified at 1 h intervals for 6 h.

### Evaluation of cytokines and nitric oxide production

Briefly, macrophages obtained from peritoneal cavity were plated at 1x10^5^ cells/mL in a 24 well plate with complete RPMI 1640 medium supplemented with 10% HIFCS and infected overnight with amastigotes of *L*. *infantum* (ratio 10:1). After 18 hours of infection, macrophages were co-cultured with splenocytes obtained from BALB/c mouse at a ratio 1:6. Cells were treated with a non-toxic concentration of CBP (100 μM) for 48 h. Afterwards, the cell supernatant was analyzed for IL-6, IL-10, monocyte chemotactic protein-1 chemokine (MCP-1), IFN-γ and TNF-α levels using flow cytometry and the Cytometric Beads Array (CBA) kit (BD Bioscience, San Diego, CA, USA). Controls consisted of sham-treated macrophages infected with *L*. *infantum*, LPS (1 μg/mL) and concanavalin A (1 μg/mL), and the negative control (macrophages without the drug). The nitric oxide content was colorimetrically determined by the Griess reaction in the culture supernatants of peritoneal macrophages [17]. Cells were treated for 24 h with cyclobenzaprine at 12 μM, corresponding to the IC_50_ value against intracellular amastigotes. Bacterial lipopolysaccharide (LPS) was used as positive control (1 μg/mL). The obtained results were extrapolated from a standard curve prepared with NaNO_2_ at different concentrations (0 to 400 μM).

#### Statistical analysis

Results are represented as the mean and standard deviation of replicates samples from at least two independent assays. IC_50_ values were calculated using sigmoidal dose-response curves using GraphPad Prism 5.0 software. Test T was used for significance testing (p<0.05).

## Results

### *In vitro* activity

**Fig 1:**
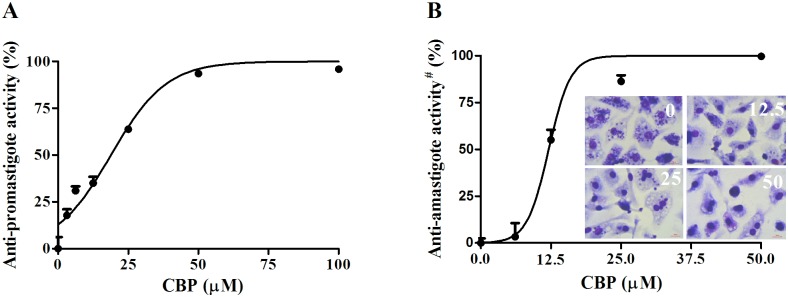
*In vitro* activity of CBP. (A) Promastigotes of *L*. *infantum* were incubated with CBP for 72 h. The growth inhibition was measured using resazurin. (n = 3) (B) Peritoneal macrophages were infected with *L*. *infantum* and treated with CBP for 72 h. The slides were stained and the results were expressed as an infection index^#^ [II = % infected cells × (number of amastigotes/total number of macrophages)]. The inset shows representative photos from the slides. (n = 3).

CBP demonstrated a concentration-dependent activity in *L*. *infantum* promastigotes growth, with an IC_50_ value of 14.5±1.1μM and an IC_90_ value of 74.5±1.2 μM (Fig 1A). The drug was also effective against clinically relevant forms, the intracellular amastigotes, with an IC_50_ of 12.6±1.05 μM and an IC_90_ of 28.7±1.3 μM (Fig 1B). The treatment with 50 μM reduced this index by 99% when compared to control (insert Fig 1B).

### Experimental visceral leishmaniasis

**Fig 2:**
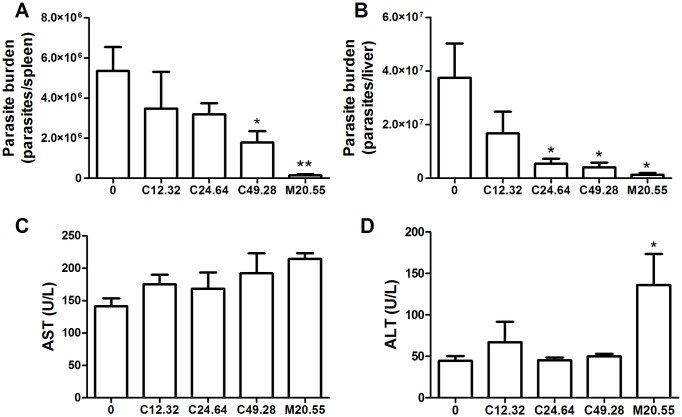
*In vivo* activity of CBP. *L*. *infantum*-infected BALB/c mice were treated after 25 days of infection for 5 consecutive days, with CBP 12.32, 24.64 and 49.28mg/kg, by oral route. Control groups were treated with miltefosine 20.55mg/kg (reference drug—M20.55) and vehicle (water). The parasite load was estimated using a parasite-limiting dilution assay (LDA) in spleen (A) and liver (B). The serum levels of aspartate aminotransferase (AST) (C) and alanine aminotransferase (ALT) (D) after five days of treatment were measured using laboratory colorimetric diagnostic kits (n = 5) *p<0.05; **p<0.01.

Cyclobenzaprine was effective in reducing the parasite load by 40.4±10.3% and 66.7±10.5% in spleen at 24.64 and 49.28 mg/kg, respectively (Fig 2A) and by 85.6±5.0 and 89.3±4.8% in liver at 24.64 and 49.28mg/kg (Fig 2B), after a short-term treatment. We did not observe changes in hepatic transaminases levels in groups treated with CBP. However, there was a significant increase of ALT level in the group treated with 20.55 mg/kg of miltefosine.

### Biochemical alterations of cyclobenzaprine on *L*. *infantum*

**Fig 3:**
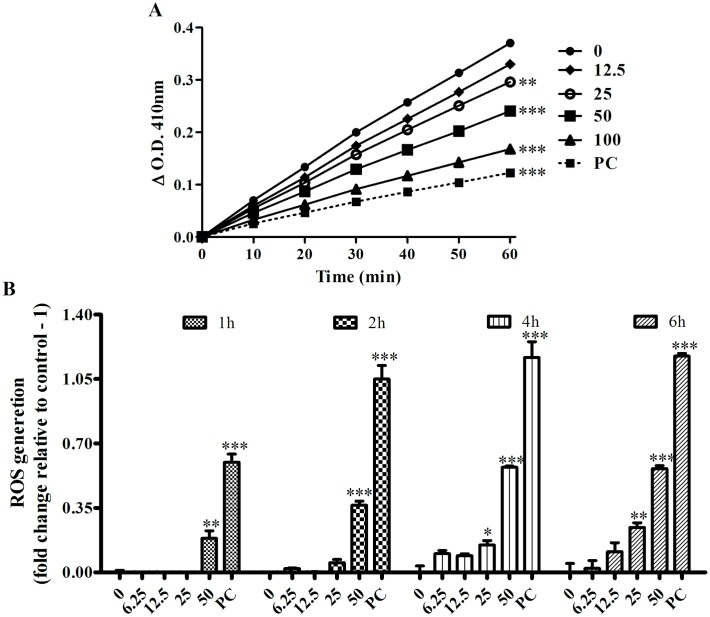
Effect of CBP on TryR and ROS production. (a) *TryR assay*. Soluble extract of promastigotes of *L*. *infantum* using oxidized trypanothione as substrate and clomipramine (50 μM) as positive control (PC) of inhibition. Reading was initiated after adding 100 μM of DTNB at 410nm (n = 3). (b) *ROS production*. Promastigotes of *L*. *infantum* were incubated with CBP and ROS generation was measured with H_2_DCFDA reagent. Antimycin A 10 μM was used as positive control (PC). (n = 3) *p<0.05, ** p<0.01 and ***p<0.001.

CBP inhibited the TryR activity in a concentration-dependent manner (Fig 3A), with a Ki of 86 ± 7.7 μM. CPB also increased the ROS production in promastigotes in a time and concentration-dependent manner in the first 6 hours, consistent with TryR inhibition (Fig 3B).

### Immunomodulatory activity

**Fig 4:**
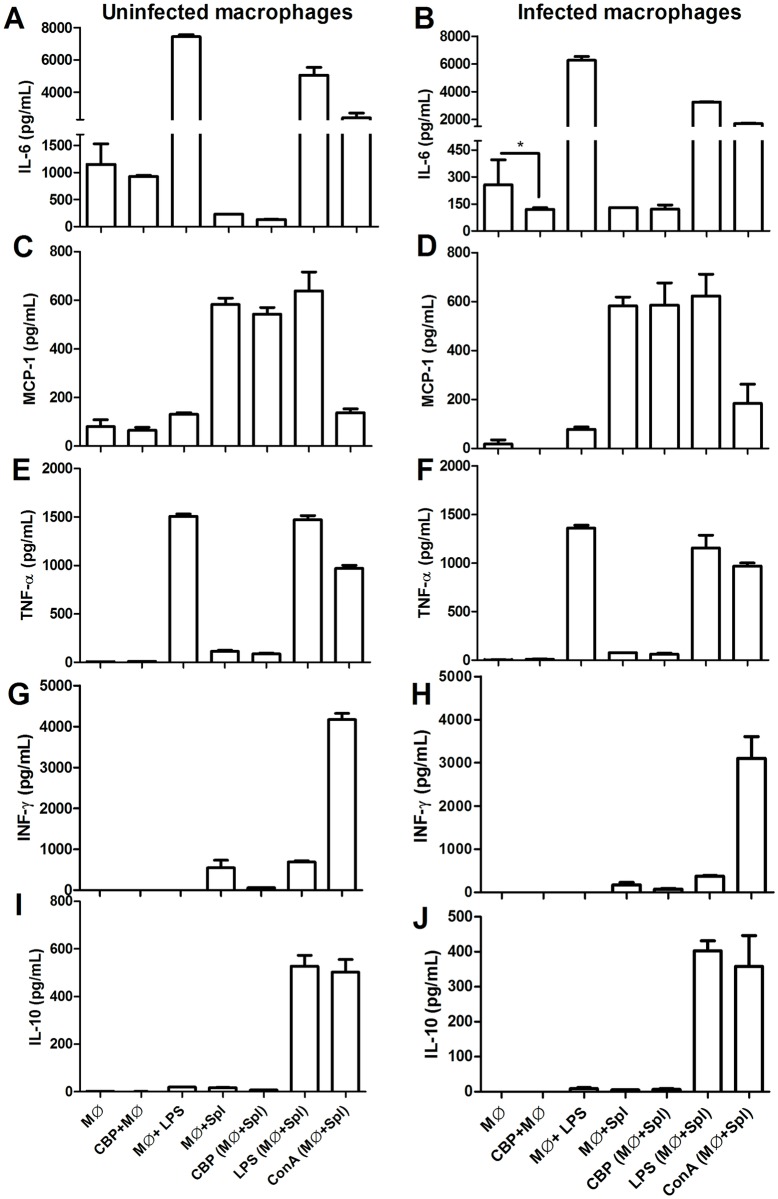
In vitro immunomodulation of CBP. *Leishmania*-infected (B, D, F, H and J) and uninfected macrophages (A, C, E, G and I), co-cultured or not with splenocytes and treated with 100 μM of CBP; MØ (macrophages), Spl (splenocytes); LPS (lipopolysaccharide); Cona (concanavalin A). Cytokine levels were measured by CBA (cytometric beads array—BD Biosciences) in supernatant of cells after 48 h. Spl were not infected with *Leishmania*. (A and B) IL-6, (C and D) MCP-1, (E and F) TNF-α, (G and H) INF-γ and (I and J) IL-10. Cytokine levels were expressed as a significant experiment of two independent assays (n = 2). *p<0.05.

**Fig 5:**
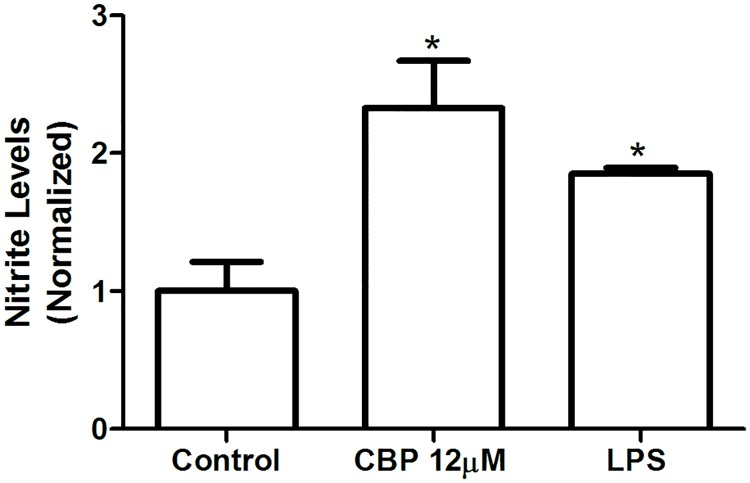
Increase of Nitric Oxide levels in macrophages treated with CBP. The production of nitric oxide (NO) by macrophages was measured after 24 h incubation with cyclobenzaprine (12 μM). The nitric oxide content was colorimetrically determined by the Griess reaction in the culture supernatants. Lipopolysaccharide (LPS) was used as positive control (1 μg/mL) (C). (n = 3) *p<0.05.

Flow cytometry data demonstrated that CBP inhibited the production of IL-6 in infected macrophages, decreasing the production of this cytokine by 53% when compared to untreated macrophages (Fig 4a and 4b). MCP-1 levels of macrophages co-cultured with splenocytes were not altered by CBP treatment (Fig 4c and 4d). The control of cytokines production was confirmed in the LPS and ConA-treated groups. The treatment of macrophages with CBP resulted in unaltered levels of TNF-α, IFN-γ and IL-10 (Fig 4e–4j). Griess assay demonstrated an upregulation of nitric oxide levels of 3.2 times in macrophages incubated with CBP (Fig 5).

## Discussion

Tricyclic antidepressants have shown potential against different *Leishmania* species with effectiveness based on the three ring structure and potency relaying on specific molecular substitutions. Amitriptyline, which lacks the nitrogen in the central ring, is 10-fold more active against promastigotes of *L*. *donovani* than its nitrogen-substituted derivative, imipramine [18]. In this work we demonstrated for the first time in literature the in vitro and in vivo antileishmanial potential of cyclobenzaprine, a tricyclic derivative structurally related to amitriptyline. Our data demonstrated that CPB was effective against the intracellular amastigotes of *L*. *infantum*, with a IC_50_ value close to that reported for amitriptyline for *L*. *donovani* and *L*. *amazonensis* intracellular amastigotes [19].

Trypanothione reductase is an essential component of the antioxidant defenses of trypanosomatids [5]. Interestingly, some tricyclic antidepressants have been reported to inhibit the TryR, as clomipramine, which showed a potent effect with a Ki of 6.53 μM on recombinant TR from *Trypanosoma cruzi* [14]. Here, we demonstrated that CBP also inhibited *L*. *infantum* TryR activity at similar Ki value to those reported for amitriptyline [14], but with a reduced affinity when compared to clomipramine. Furthermore, it was also observed that promastigotes treated with CBP increased the intracellular concentration of ROS, in the same concentration range for TryR inhibition. Nevertheless, the concentration of CBP required to inhibit TryR and to induce ROS accumulation in the first hours of incubation was higher to that required to eliminate the parasites after 72 h. These data suggest a cumulative effect of inhibition of TryR at lower concentrations of CBP in 72 h or concomitant off-target effects of CBP contributing to the parasite death.

Immunomodulatory activity of drugs has also been implicated in the parasite control. In addition to the effective antileishmanial activity observed for CBP, its immunomodulatory potential was evaluated. Considered as a positive effect for *Leishmania* control, our data demonstrated that CPB induced a decrease of IL-6 levels in *Leishmania*-infected macrophages. Cytokines such as IL-6 and IL-10 play a critical role in regulating macrophage activation. Several clinical and experimental studies show that both IL-6 and IL-10 are involved in pathogenesis of VL; elevated levels of IL-6 has been associated to a preceding death event in VL patients [20]. It has also been reported that patients with active VL have higher serum levels of IL-10 and IL-6, evidencing their association with disease persistence [21]. Other studies with phenylpropanoid derivatives, demonstrated a correlation between the downregulation of IL-6 and IL-10 with the control of the infection in the *Leishmania*-infected macrophages [22]. Jek and co-workers [23] demonstrated that blocking NO production in rat macrophages results in an increased IL-1 and IL-6 secretion. Our assays demonstrated an upregulation of NO levels in macrophages incubated with CBP. The potential anti-inflammatory effect of nitric oxide has been described in human alveolar macrophages, followed by a downregulation of proinflammatory cytokines, including IL-6 [24, 25]. Based in these data, we could hypothesize that the antileshmanial effect of CBP involves an immunomodulatory effect in macrophages, increasing NO levels which consequently reduces an exacerbatory cytokine (IL-6) in leishmaniasis.

## Conclusion

To the best of our knowledge, this study is the first report of the *in vitro* and *in vivo* antileishmanial activity of the FDA-approved drug CBP. Modulation of macrophage immune response and induction of oxidative stress in parasite seem to contribute to this efficacy, but additional effects may be further investigated. Altogether, these findings point out CBP as a candidate for further studies against visceral leishmaniasis.
